# Individual- and Facility-Level Factors Associated with Facility Testing among Men in Malawi: Findings from a Representative Community Survey

**DOI:** 10.3390/diagnostics11060950

**Published:** 2021-05-26

**Authors:** Kelvin Balakasi, Brooke E. Nichols, Misheck Mphande, Christian Stillson, Shaukat Khan, Pericles Kalande, Isabella Robson, Maria Sanena, Khumbo Ng’ona, Joep J. van Oosterhout, Naoko Doi, Kathryn Dovel

**Affiliations:** 1 Partners in Hope, Lilongwe, Malawi; misheck@pihmalawi.com (M.M.); pkalande@pihmalawi.com (P.K.); iserobson@gmail.com (I.R.); maria@pihmalawi.com (M.S.); joep@pihmalawi.com (J.J.v.O.); KDovel@mednet.ucla.edu (K.D.); 2 Department of Global Health, School of Public Health, Boston University, Boston, MA 02118, USA; Brooken@bu.edu; 3 Health Economics and Epidemiology Research Office, Department of Internal Medicine, School of Clinical Medicine, Faculty of Health Sciences, University of the Witwatersrand, Johannesburg 2000, South Africa; 4 Clinton Health Access Initiative, Boston, MA 02127, USA; cstillson@clintonhealthaccess.org (C.S.); skhan@clintonhealthaccess.org (S.K.); naoko.d@gmail.com (N.D.); 5 Department of HIV/AIDS, Malawian Ministry of Health, Lilongwe, Malawi; khumbongona7@gmail.com; 6 Division of Infectious Disease, David Geffen School of Medicine, University of California Los Angeles, Los Angeles, CA 90095, USA

**Keywords:** men, HIV testing, PITC, facility visits, OPDs, sub-Saharan Africa, Malawi

## Abstract

(1) Background: Men frequent outpatient departments (OPD) but are underrepresented in HIV testing services throughout sub-Saharan Africa. (2) Methods: We conducted a secondary analysis on data from a community-based survey with men in rural Malawi to assess factors associated with HIV testing, and being offered testing, during men’s OPD visits. We include OPD visits made by men in-need of testing as our unit of observation. Multilevel mixed-effects logistic regression models were conducted. (3) Results: 782 men were eligible for these analyses, with 1575 OPD visits included (median two visits per man; IQR 1–3). 17% of OPD visits resulted in HIV testing. Being offered testing (aOR 42.45; 95% CI 15.13–119.10) and satisfaction with services received (aOR 3.27; 95% CI 1.28–8.33) were significantly associated with HIV testing. 14% of OPD visits resulted in being offered HIV testing. Being married/steady relationship (aOR 2.53; 95% CI 1.08–5.91) and having a sexual partner living with HIV (aOR 8.22; 95% CI 1.67–40.49) were significantly associated with being offered testing. (4) Conclusion: Being offered HIV testing was the strongest factor associated with testing uptake, while HIV status of sexual partner had the strongest association with being offered testing. Implementation of provider-initiated-testing should be prioritized for male OPD visits.

## 1. Introduction

Men are underrepresented in HIV testing services throughout sub-Saharan Africa [1,2]. HIV testing is critical to achieve the UNAIDS 95-95-95 goals [3], as it is the first entry point to HIV care. In Malawi, an estimated 14% of HIV positive men are undiagnosed, while only 6% of HIV positive women remain undiagnosed [2]. Improved HIV testing among men is key to reaching UNAIDS goals, and to curbing HIV epidemics in the region.

HIV testing coverage among men could be improved through facility-based, provider-initiated testing and counseling (PITC). Across the continent, PITC remains the primary strategy for identifying individuals living with HIV [4,5], and a recent study suggests PITC among guardians (i.e., non-clients attending facilities for someone else’s care) comprises a major portion of individuals identified as living with HIV [6]. New evidence from Malawi suggests that the majority of men in need of HIV testing (defined as never tested for HIV or tested >12 months ago, per Malawi Ministry of Health guidelines) [7] frequently attend outpatient departments (OPD) for routine health services [8], but rarely test for HIV when at OPD [9,10,11,12,13,14]. OPD is one of few entry points for men into the health care system and HIV services more specifically [15,16], making OPD testing, and understanding factors associated with men’s testing at OPD, critical to future HIV diagnostic efforts among men. 

Most of the literature on factors associated with HIV testing for men focus on generalized populations, not those who are already at the OPD receiving routine services. Generalized barriers to testing include transportation costs, time required to travel to facilities and wait for services, and fear of lack of privacy and confidentiality [15,17,18,19]. However, men already at the health facility may not experience these same barriers since they have already overcome challenges related to transportation and time. The few surveys that have been conducted suggest that, at the individual-level, fear of an HIV positive test result and fear of unwanted disclosure and stigma may discourage OPD testing [17,18,19,20]. At the facility-level, long wait-times, poor fidelity to PITC guidelines by health care workers (HCWs), and lack of privacy at HIV testing sites may discourage uptake of testing among men already at the health facility [10,21,22,23]. 

In this study, we conduct a secondary analysis on data from a representative community-based survey with men in rural Malawi to assess individual- and facility-level factors associated with HIV testing, as well as being assessing offers of PITC, during OPD visits attended by men in-need of testing.

## 2. Materials and Methods 

### 2.1. Setting, Design

We conducted a secondary analysis on data from a cross-sectional, representative community survey with men from 36 villages in central and southern Malawi. For the parent study, household census listings were used to randomly select eligible respondents using computer randomized number generation. Eligibility criteria for the parent study were: (1) aged 15–64 years; (2) current resident of the participating village; (3) spent >15 nights within the village in the past 30 days; and (4) never tested HIV-positive. Random selection within each village was stratified by age categories: young men (15–24-years, *n* = 300); middle-aged men (25–39-years, *n* = 425); and older men (40+-years, *n* = 425). Detailed information on design and data collection of the main study can be found elsewhere [6]. For this sub-study, we included men who attended at least one OPD clinic visit in the past 24 months and were in-need of HIV testing during at least one of those OPD visits. We defined in-need of HIV testing as never tested or tested >12 months prior to the observed OPD visit, AND never tested HIV-positive prior to the observed OPD visit.

### 2.2. Data and Methods

The study team recruited randomly selected individuals with the assistance of community health workers and village chiefs. Surveys were conducted with all eligible men. Survey domains included: (1) sociodemographic characteristics; (2) self-reported HIV testing history, defined as ever tested HIV-positive, tested HIV-negative in the past 12 months, or in-need of testing; and (3) a detailed description of the last four facility visits made in the past 24 months; including services received, satisfaction with services received, facility characteristics that may be associated with HIV testing, testing during each clinic visit, and reasons for not testing. Surveys lasted approximately 55 minutes on average and were conducted in the local language (Chichewa) by trained, male research assistants.

#### Variables

For this paper, we used each OPD visit by a man in-need of testing at the time of the visit as a unique observation. We included all OPD visits made in the past 24 months, meaning that individual men could contribute multiple OPD visit observations to the dataset. Each OPD visit was analyzed as a separate unit of observation, even if they came from the same respondent. We categorized each OPD visit into either guardian or client visits—client visits were defined as any visit whereby the primary health service received was for the respondent (i.e., visit was for men’s own health care). Guardian visits were defined as visits primarily supporting the health services of someone else (i.e., visit was for a family member or friend’s health care). 

Our independent factors are grouped into individual-level factors and facility level factors. Individual-level factors are categorized largely into (1) demographic factors (age, marital status, schooling, employment and household wealth quantile) and, (2) sexual behavior and HIV perception factors (sexual risk, sexual partner HIV status and perceived stigma from testing HIV positive). Facility-level factors are categorized into (1) services received (facility type, distance to the health facility and whether the visit was primarily as a guardian), (2) quality of care (offering of HIV testing and satisfaction with services received) and (3) facility type where OPD visits were made (public facility owned and managed by the government and offering free services, private for pay facility, or mission hospitals that require consultation fees but offer free HIV testing). We define sexual risk as having sex without a condom with an unstable partner or having more than two sexual partners in the past 12-months. Perceived stigma and satisfaction with services received variables were measured using a 4-point Likert scale. We dichotomized the responses in this analysis. We calculate distance to the facility using the Euclidian distance between the GPS coordinates of the respondent’s home (collected during the interview) and the GPS coordinates of the facility the respondent visited. Respondents were allowed to provide multiple responses for not testing for HIV during an OPD visit.

### 2.3. Analysis

We examined associations of individual- and facility-level factors with HIV testing at any given clinic visit. We used a series of nested multilevel mixed-effects logistic regression models to examine factors associated with HIV testing at the individual-, visit characteristics- and quality of care-levels. We also conducted a secondary analysis with the same population to assess individual- and facility-level factors associated with being offered HIV testing, since being offered testing is a critical step towards actual testing. For each analysis, we used nested models to assess if and how associations change within each level when new levels are added. 

We consider random effects from clustering of observations at two-levels: clinical visits clustered within an individual and individuals clustered within a village. We did not control for clustering at the facility level because the number of observations (OPD visits) was too small for some clusters. Robust standard errors are estimated in each model to control for any additional clustering. All analyses were completed in Stata v.15.

### 2.4. Human Subjects 

The parent study was approved by the National Health Sciences Review Committee of Malawi (IRB#2338, 9 August 2019) and the University of California Los Angeles Institutional Review Board (IRB#17-000109). Written consent was ascertained from all respondents and written assent was attained for respondents aged between 15–18 years.

## 3. Results

1116 men completed a survey in the parent study between 17 August 2019 and 18 September 2019. Among these, 782 men had at least one eligible OPD visit and were included in these analyses. 79.7% of men were married, the median age was 33 years (IQR: 22–42), the median duration of schooling was 5.9 years (SD = 3.4), and they were evenly distributed across the wealth quantiles. Men had a median of two OPD visits in the past 24-months (IQR: 1–3). 43.9% of men reported having been tested for HIV at any point during the past 24-months (Table 1).

### 3.1. Characteristics of the OPD Visits

In total, men included in this analysis made 1575 eligible OPD visits, whereby the respondent was in need of HIV testing during the OPD visit (Table 2). Most of the OPD visits were made to a publicly owned health facility (80.2%), with 38.9% of the visits made as a guardian (not a client). On average, men traveled a mean distance of 16.0 km (SD = 15.0 km) for each OPD visit. Men tested for HIV at 16.7% of all OPD visits whist offering of HIV testing happened during 14.4% of all OPD visits.

### 3.2. Factors Associated with HIV Testing at an OPD Visit

Among all OPD visits made by men in-need of HIV testing, 17% of visits resulted in same-day HIV testing, the proportion of men sought testing at their own initiative (VCT). Table 3 examines factors associated with same-day HIV testing at OPD visits, using multi-level multivariate logistic regressions. Model 1 assessed individual-level factors associated with testing and found that being married or having a steady partner (aOR 1.96; 95% CI 1.11–3.45) and having a known HIV+ sexual partner (aOR 3.4; 95% CI 1.04–11.06) were significantly associated with a higher likelihood to be tested for HIV during OPD visits. In contrast, OPD visits made by men in the lowest wealth quantile (aOR 0.78; 95% CI 0.61–0.99) or the highest wealth quantile (aOR 0.72; 95% CI 0.54–0.95) were less likely to result in same-day HIV testing than visits by men in the middle-wealth quantile.

In Model 2, we added visit characteristics and found that individual-level associations from Model 1 were sustained when including facility type, distance to the health facility and visit type (guardian versus client). OPD visits to a privately-owned health facility (aOR 0.21; 95% CI 0.07–0.66) and guardian visits (as opposed to a client visits) (aOR 0.25; 95% CI 0.16–0.38) were both negatively associated with same-day HIV testing. 

Model 3 incorporated quality of care variables into the full model. When adding quality of care, all association of individual-level variables with HIV testing lost significance. Quality of care variables had the strongest associations with HIV testing. Being offered HIV testing had the highest positive association with same-day testing during an OPD visit (aOR 42.45; 95% CI 15.13–119.10). Satisfaction with services received also had a positive association (aOR 3.27; 95% CI 1.28–8.33). OPD visits made to private facilities and as guardians remained significantly and negatively associated with testing. 

#### Reasons for Not Testing for HIV at a Clinic Visit

Table 4 shows self-reported reasons for men not testing during OPD visits. Not being offered HIV testing was the most common reason reported for not testing (40%), followed by low perceived risk of HIV (27%) and not being prepared to test (20%). Significant differences inn reasons cited for not testing among clients and guardians were not being offered HIV testing (clients 45.7% vs. guardians 32.5%) and privacy concerns (clients 1.6% vs. guardians 0%).

### 3.3. Factors Associated with Being Offered HIV Testing at an OPD Visit

In Table 5, we examined factors associated with being offered HIV testing during an OPD visit. We focus on this since it had by far the strongest association with same-day testing, and was the most common reason cited for not testing for HIV at an OPD visit. In Model 1, married or having a steady partner (aOR 2.30; 95% CI 1.02–5.20) and having a sexual partner with a known HIV+ status (aOR 6.82; 95% CI 1.33–34.93) were positively associated with being offered HIV testing. With the addition of ‘services received’ factors in Model 2, the association with having a sexual partner with a known HIV+ status remained significant (aOR 8.31; 95% CI 1.67–41.29). Guardian visits (aOR 0.46; 95% CI 0.37–0.57) and OPD visits to a privately-owned health facility (aOR 0.28; 95% CI 0.09–0.85) are negatively associated with being offered HIV testing.

In Model 3, the addition of satisfaction with services received did not affect the associations observed in Model 2.

## 4. Discussion

Using self-reported survey data, we found that 17% of OPD visits made by men in need of HIV testing in Malawi resulted in HIV testing. Being offered HIV testing by a health care worker was the primary factor associated with actual HIV testing uptake, with weaker associations with satisfaction with quality of services received at OPD, accessing care from public facilities, and attending the OPD as a client (versus guardian). Importantly, all individual-level variables fall out of significance when including ‘offered HIV testing’ in the model, suggesting that simply improving the fidelity and reach of PITC for men visiting OPD could drastically improve testing coverage among men. Interventions to increase PITC for men should especially focus on men visiting the OPD as guardians and at private facilities.

In our study, only 14% of OPD visits made by men in need of HIV testing resulted in being offered HIV testing (17% of client visits and 11% of guardian visits). Being married or in a stable relationship and having a partner with a known HIV-positive status were associated with being offered testing. Like other African countries, Malawi has adopted national guidelines that PITC should be provided during all facility visits where the client is in need of testing [2,21]. However, we find that there is poor fidelity in implementation of these guidelines. Numerous other studies in the region find similarly low rates of PITC, ranging from 4–28% of eligible OPD clients offered HIV testing [9,10,11,12,13,14]. This is not surprising given the multiple barriers to PITC experienced within OPD clinics, such as overburdened health care workers, lack of infrastructure for private testing spaces, and a high volume of OPD clients at any given time. In private facilities, reasons for poor implementation might be different. Health care workers know that often individuals do not like to be confronted with HIV testing and implementing PITC could affect client satisfaction which is critical to clients coming back and paying for services. 

Our findings will not surprise those familiar with PITC efforts in OPD settings, but are critical given recent shifts in global HIV testing priorities. Donors such as PEPFAR have recently de-emphasized facility-based OPD testing due to low positivity rates, with a major push toward targeted, higher-yield strategies [5], yet such shifts may be in direct conflict with global and national goals to increase testing among men. Our findings suggest that simple, scalable solutions of PITC within OPD settings are critical to reaching men with HIV testing, and should not be overlooked. Other literature corroborates our findings, showing that low coverage of PITC at OPD may substantially affect men’s overall uptake of HIV services [12,17,20]. The fact that the majority of men in-need of testing eventually attend OPD clinics [8] highlights the important role that PITC can play to reach men. Generalized PITC for men attending OPD should remain a priority activity, otherwise we will miss critical opportunities to test and link men to HIV services. Additional research is needed to identify effective strategies that target PITC toward high-risk men, although to date efforts have not been successful [24,25].

Our data on self-reported reasons for not testing during an OPD visit suggest that men who overcome initial barriers to general facility visits may not face the same barriers to HIV testing as seen in community-based male populations. Very few men reported that fear of perceived stigma, distance to a health facility, or time requirements to complete a test kept them from testing, all of which are established barriers to testing and often limit men’s use of general health services [15,17,18,19,21]. 

Several studies have shown an association between HIV testing and sociodemographic characteristics such as age, education, household wealth and being married [24,25,26,27]. Our study only found association with marital status and household wealth. We hypothesize that while these socio-demographic characteristics might significantly affect a man’s decision to go for an HIV test, they do not have a significant bearing on a man’s decision to test when already at the facility, especially if medical personnel recommend HIV testing during routine care. 

Importantly, we see no difference in HIV testing uptake among men who report 2+ sexual partners, condomless sex, or having a sexual partner known to be living with HIV. A study from South Africa also showed no difference in testing uptake between men with high- and low-risk profiles [28]. Our finding may represent men’s misconceptions regarding risk of HIV, the fact that HIV and HIV testing is not a primary concern for men attending OPD clinics, or simply the fact that men at OPD largely only test when offered testing, regardless of risk factors. Combined, these findings suggest missed opportunities to test higher-risk men who are already at the health facility.

Finally, our study showed that satisfaction with services received was associated with HIV testing, similarly to other studies [18,20,29,30]. Interactions with medical personnel can shape perceptions of patients, including decisions to seek further health services that were not the primary focus for the clinic visit. Provision of good quality services combined with offering PITC at OPD could therefore encourage more men to access HIV testing.

This study is subject to several limitations. First, survey data rely on self-report and may be susceptible to recall and social desirability bias. In particular, men who did not test for HIV may not remember being offered HIV testing, therefore there is a risk that we overestimate the relationship between being offered testing and actual HIV testing. The effect size was sufficiently large that we do not expect this limitation to explain the relationship. Second, we had limited data on the reason for each clinic visit, which may be associated with being offered HIV testing. Finally, we have limited data on satisfaction with services received. More robust measures of satisfaction, and satisfaction with specific aspects of services received, may provide more nuanced analyses.

## 5. Conclusions

Malawian men in need of HIV testing frequently visited OPD clinics as client or guardian, but just 17% of men’s OPD visits resulted in HIV testing. Being offered HIV testing was the strongest predictor of same-day HIV testing; however, only 14% of OPD visits resulted in the man being offered testing. Interventions that focus on improving fidelity of PITC strategies, or implementation of novel facility-based HIV testing strategies, may improve testing uptake among men.

## Figures and Tables

**Table 1:** Socio-demographic features of men who attended an OPD visit in the past 24-months and were eligible for HIV testing (*n* = 782).

Variables	Total *n* = 782	Youth (15–24 Years) *n* = 225	Middle Aged (25–39 Years) *n* = 306	Older Men (40+ Years) *n* = 251
Demographics				
Age, median (IQR)	33 (22–42)	18 (16–21)	32 (27–36)	47 (43–55)
Married/steady partner, % (*n*)	79.7 (623)	35.1 (79)	97.1 (297)	98.4 (247)
Lives with a child aged ≤ 2 years, % (*n*)	28.0 (219)	7.6 (17)	46.4 (142)	23.9 (60)
Years of schooling, mean (SD)	5.9 (3.4)	6.5 (2.8)	6.3 (3.5)	4.9 (3.4)
Household wealth quantile, mean (SD)				
1 poor	32.9 (257)	36.0 (81)	39.2 (120)	22.3 (56)
2 middle income	35.4 (277)	33.8 (76)	36.3 (111)	35.9 (90)
3 rich	31.7 (248)	30.2 (68)	24.5 (75)	41.8 (105)
Sexual behavior and HIV perceptions				
High sexual risk behaviour ^, % (*n*)	35.3 (276)	36.9 (83)	38.6 (118)	29.9 (75)
Sexual partner known HIV+, % (*n*)	0.9 (7)	0.4 (1)	0.7 (2)	1.6 (4)
High perceived likelihood of HIV+ test stigma, % (*n*)	31.7 (248)	40.9 (92)	29.7 (91)	25.9 (65)
Number of health facility visits in ≤ 24 months, median (IQR)	2 (1–3)	2 (1–2)	2 (1–3)	2 (1–3)
Tested for HIV in the past 24-months, % (*n*)	43.9 (344)	30.2 (68)	54.9 (168)	43.0 (108)

^ Defined as having condomless sex with an unstable partner or having two or more sexual partners.

**Table 2:** Characteristics of OPD visits among men in Malawi (*n* = 1575).

Variables	Total *n* = 1575	Youth (15–24 Years) *n* = 225	Middle Aged (25–39 Years) *n* = 306	Older Men (40+ Years) *n* = 251
Services received				
Facility type, % (*n*)				
Public	80.2 (1263)	83.7 (361)	79.9 (504)	77.6 (398)
Private	4.7 (74)	4.9 (21)	4.3 (27)	5.1 (26)
CHAM	15.1 (238)	11.4 (49)	15.8 (100)	17.3 (89)
Distance to health facility (in km), mean (SD)	16.0 (15.0)	15.2 (14.9)	16.9 (15.4)	15.6 (14.6)
Visit type, guardian (vs. client visit), % (*n*)	38.9 (613)	25.1 (108)	48.0 (303)	39.4 (202)
Quality of care				
Offered HIV testing, % (*n*)	14.4 (226)	11.8 (51)	15.4 (97)	15.2 (78)
Satisfied with service received * % (*n*)	90.4 (1424)	90.3 (389)	89.2 (563)	92.0 (472)
HIV services				
Tested for HIV, % (*n*)	16.7 (263)	12.5 (54)	18.4 (116)	18.1 (93)

* For guardians, satisfaction was defined as satisfaction with OPD services received by the client they escorted/cared for.

**Table 3:** Multivariate logistic regression of factors associated with HIV testing at an OPD visit.

Variables	Model 1 aOR (95% CI)	Model 2 aOR (95% CI)	Model 3 aOR (95% CI)
Individual level			
Demographics			
Respondent age category			
Youth (15–24 years)	0.96 (0.56–1.65)	0.82 (0.45–1.49)	0.73 (0.32–1.70)
Middle aged (25–39 yrs)	Ref	Ref	Ref
Older men (40+ years)	0.96 (0.72–1.27)	0.89 (0.66–1.19)	0.82 (0.49–1.37)
Married/steady partner	1.96 * (1.11–3.45)	2.24 ** (1.26–3.99)	2.06 (0.88–4.80)
Lives with a child aged ≤ 2 years	1.04 (0.83–1.30)	1.11 (0.88–1.39)	1.26 (0.79–1.99)
Years of schooling	0.97 (0.92–1.02)	0.96 (0.92–1.01)	0.96 (0.91–1.02)
Household wealth quantile			
1 poor	0.78 * (0.61–0.99)	0.71 ** (0.55–0.91)	0.64 (0.40–1.03)
2 middle income	Ref	Ref	Ref
3 rich	0.72 * (0.54–0.95)	0.72 * (0.55–0.96)	0.79 (0.49–1.26)
Sexual behavior and HIV perceptions			
High sexual risk behaviour ^	0.85 (0.69–1.05)	0.86 (0.68–1.07)	0.82 (0.57–1.19)
Sexual partner known HIV+	3.40 * (1.04–11.06)	4.06 * (1.25–13.24)	1.40 (0.34–5.85)
High perceived likelihood of HIV+ test stigma	0.90 (0.69–1.18)	0.84 (0.61–1.15)	0.89 (0.57–1.39)
Visit characteristics			
Facility type			
Public		Ref	Ref
Private		0.21 ** (0.07–0.66)	0.20 * (0.06–0.70)
CHAM		1.21 (0.81–1.81)	0.99 (0.58–1.67)
Distance to health facility (in km)		1.01 (0.99–1.02)	0.99 (0.98–1.01)
Visit type, guardian (vs. client visit)		0.25 ** (0.16–0.38)	0.18 ** (0.08–0.39)
Quality of care			
Offered HIV testing			42.45 ** (15.13–119.10)
Satisfied with service received			3.27 * (1.28–8.33)

^ Defined as having condomless sex with an unstable partner or having two or more sexual partners. * *p* < 0.05, ** *p* < 0.01.

**Table 4:** Reasons for not testing for HIV during an OPD visit (*n* = 1499).

Reason for Not Testing	Total *n* = 1499	Client Visits *n* = 895	Guardian Visits *n* = 604	*p*-Value ^
Individual level				
Low perceived HIV risk, % (*n*)	27.0 (405)	27.4 (245)	26.5 (160)	0.705
Not ready/prepared to test for HIV, % (*n*)	19.8 (296)	18.6 (166)	21.5 (130)	0.156
Occupied with guardian responsibilities, % (*n*)	5.7 (86)	NA	14.2 (86)	-
Other reasons ^a^, % (*n*)	0.4 (6)	0.3 (3)	0.5 (3)	0.690
Facility level				
Not offered HIV testing by provider, % (*n*)	40.4 (605)	45.7 (409)	32.5 (196)	0.000 **
Time required to access HIV testing, % (*n*)	4.7 (71)	5.5 (49)	3.6 (22)	0.101
Service not accessible ^b^, % (*n*)	1.1 (16)	1.0 (9)	1.2 (7)	0.777
Privacy concerns, % (*n*)	0.9 (14)	1.6 (14)	0 (0)	0.001 **

^ *p*-values calculated using Chi-squared test, except for privacy concerns, which was calculated using Fisher’s Exact test. ^a^ Included not having a health passport at the time of the clinic visit (for guardians) and not having a specific reason (for clients). ^b^ Included stock-out of testing materials, HIV testing clinic closed at the time of the visit, provider not available to offer service, or lack of funds to pay for the service. * *p* < 0.05, ** *p* < 0.01.

**Table 5:** Multivariate logistic regressions of factors associated with offered HIV testing at a clinic visit.

Variables	Model 1 aOR (95% CI)	Model 2 aOR (95% CI)	Model 3 aOR (95% CI)
Individual level			
Demographics			
Respondent age category			
Youth (15–24 years)	1.12 (0.54–2.36)	1.04 (0.48–2.24)	1.05 (0.49–2.25)
Middle aged (25–39 yrs)	Ref	Ref	Ref
Older men (40+ years)	0.95 (0.59–1.54)	0.91 (0.55–1.49)	0.91 (0.55–1.50)
Married/steady partner	2.30 * (1.02–5.20)	2.52 * (1.07–5.91)	2.53 * (1.08–5.91)
Lives with a child aged ≤ 2 years	0.91 (0.66–1.26)	0.93 (0.66–1.32)	0.94 (0.66–1.34)
Years of schooling	0.97 (0.92–1.03)	0.97 (0.92–1.02)	0.97 (0.92–1.02)
Household wealth quantile			
1 poor	0.90 (0.61–1.32)	0.85 (0.56–1.30)	0.86 (0.56–1.31)
2 middle income	Ref	Ref	Ref
3 rich	0.69 (0.47–1.01)	0.69 (0.46–1.04)	0.69 (0.45–1.04)
Sexual behavior and HIV perceptions			
High sexual risk behaviour ^	0.89 (0.63–1.24)	0.90 (0.64–1.26)	0.90 (0.64–1.27)
Sexual partner known HIV+	6.82 * (1.33–34.93)	8.31 ** (1.67–41.29)	8.22 ** (1.67–40.49)
High perceived likelihood of HIV+ test stigma	0.82 (0.56–1.18)	0.78 (0.53–1.16)	0.78 (0.53–1.16)
Services received			
Facility type			
Public		Ref	Ref
Private		0.28 * (0.09–0.85)	0.28 * (0.09–0.85)
CHAM		1.38 (0.71–2.69)	1.36 (0.69–2.67)
Distance to health facility (in km)		1.01 (1.00–1.03)	1.01 (1.00–1.03)
Visit type, guardian (vs. client visit)		0.46 ** (0.37–0.57)	0.46 ** (0.36–0.57)
Quality of care			
Satisfied with service received			1.18 (0.58–2.39)

^ Defined as having condomless sex with an unstable partner or having two or more sexual partners. * *p* < 0.05, ** *p* < 0.01.

## Data Availability

Data is not currently available on a repository. Individuals may request data from the corresponding author.
